# Famotidine inhibits toll-like receptor 3-mediated inflammatory signaling in SARS-CoV-2 infection

**DOI:** 10.1016/j.jbc.2021.100925

**Published:** 2021-06-30

**Authors:** Rukmini Mukherjee, Anshu Bhattacharya, Denisa Bojkova, Ahmad Reza Mehdipour, Donghyuk Shin, Khadija Shahed Khan, Hayley Hei-Yin Cheung, Kam-Bo Wong, Wai-Lung Ng, Jindrich Cinatl, Paul P. Geurink, Gerbrand J. van der Heden van Noort, Krishnaraj Rajalingam, Sandra Ciesek, Gerhard Hummer, Ivan Dikic

**Affiliations:** 1 Institute of Biochemistry II, Faculty of Medicine, Goethe University, Frankfurt, Germany; 2 Buchmann Institute for Molecular Life Sciences, Goethe University, Frankfurt, Germany; 3 Max Planck Institute of Biophysics, Frankfurt, Germany; 4 Institute of Medical Virology, University Hospital Frankfurt, Frankfurt, Germany; 5 Department of Theoretical Biophysics, Max Planck Institute of Biophysics, Frankfurt, Germany; 6 Department of Systems Biology, College of Life Science and Biotechnology, Yonsei University, Seoul, Republic of Korea; 7 School of Pharmacy, Faculty of Medicine, The Chinese University of Hong Kong (CUHK), Hong Kong, Hong Kong; 8 State Key Laboratory of Agrobiotechnology, School of Life Sciences, The Chinese University of Hong Kong (CUHK), Hong Kong, Hong Kong; 9 Oncode Institute and Department of Chemical Immunology, Leiden University Medical Centre, Leiden, The Netherlands; 10 Cell Biology Unit, University Medical Center of the Johannes Gutenberg University Mainz, Mainz, Germany; 11 Institute of Pharmaceutical Biology, Goethe-University, Frankfurt, Germany; 12 Fraunhofer Institute for Molecular Biology and Applied Ecology (IME), Branch Translational Medicine and Pharmacology, Frankfurt, Germany; 13 Institute of Biophysics, Goethe University Frankfurt, Frankfurt, Germany

**Keywords:** famotidine, toll-like receptor, SARS-CoV-2, antiviral signaling, histamine, CPE, cytopathic effect, DSF, dynamic scanning fluorimetry, FRET, fluorescence resonance energy transfer, GERD, gastroesophageal reflux disease, MC, mast cell, PAMP, pathogen-associated molecular pattern, PKA, protein kinase A, SARSCoV-2, severe acute respiratory syndrome coronavirus 2, TLR3, toll-like receptor 3

## Abstract

Apart from prevention using vaccinations, the management options for COVID-19 remain limited. In retrospective cohort studies, use of famotidine, a specific oral H2 receptor antagonist (antihistamine), has been associated with reduced risk of intubation and death in patients hospitalized with COVID-19. In a case series, nonhospitalized patients with COVID-19 experienced rapid symptom resolution after taking famotidine, but the molecular basis of these observations remains elusive. Here we show using biochemical, cellular, and functional assays that famotidine has no effect on viral replication or viral protease activity. However, famotidine can affect histamine-induced signaling processes in infected Caco2 cells. Specifically, famotidine treatment inhibits histamine-induced expression of Toll-like receptor 3 (TLR3) in SARS-CoV-2 infected cells and can reduce TLR3-dependent signaling processes that culminate in activation of IRF3 and the NF-κB pathway, subsequently controlling antiviral and inflammatory responses. SARS-CoV-2-infected cells treated with famotidine demonstrate reduced expression levels of the inflammatory mediators CCL-2 and IL6, drivers of the cytokine release syndrome that precipitates poor outcome for patients with COVID-19. Given that pharmacokinetic studies indicate that famotidine can reach concentrations in blood that suffice to antagonize histamine H2 receptors expressed in mast cells, neutrophils, and eosinophils, these observations explain how famotidine may contribute to the reduced histamine-induced inflammation and cytokine release, thereby improving the outcome for patients with COVID-19.

The COVID-19 pandemic caused by the severe acute respiratory syndrome coronavirus 2 (SARSCoV-2) affected more than 100 million people with more than 2 million deaths as of February 18, 2021. Many drugs are being repurposed for COVID-19 therapy, often based on insufficient evidence from randomized trials. Famotidine is an approved drug for peptic ulcers and gastroesophageal reflux disease (GERD) (Fig. 1*A*). It acts as a competitive antagonist of histamine in gastric parietal cells. Histamine activates protein kinase A (PKA), which causes movement of H+/K+ transporters to the membrane resulting in more acid secretion. Famotidine counters this activity, thereby reducing acid secretion in GERD patients.

**Figure 1:**
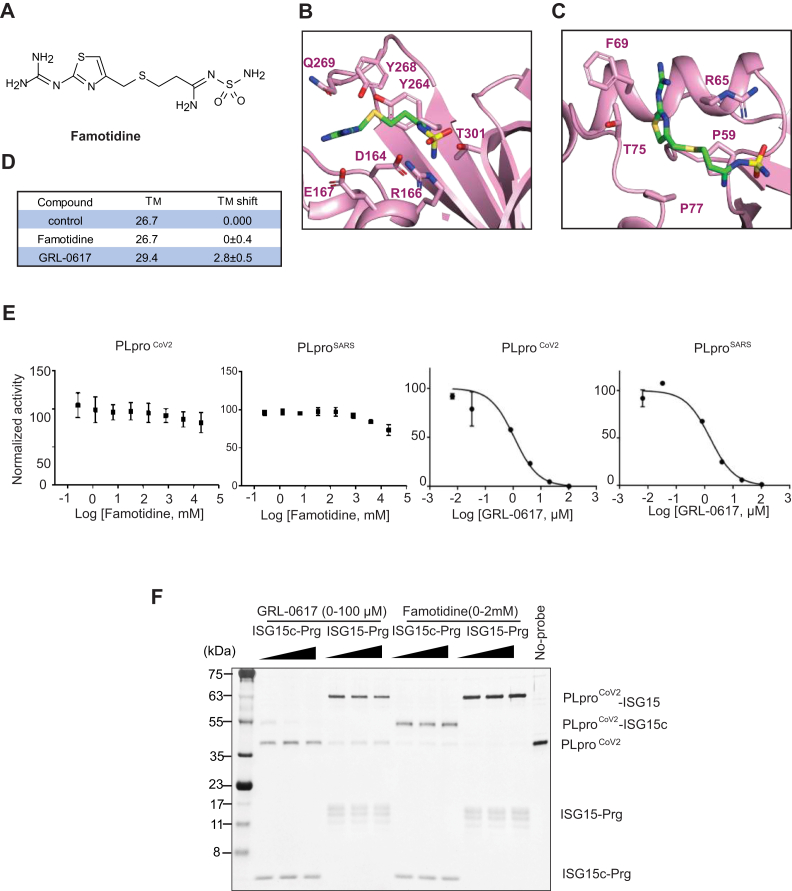
**Effect of famotidine on PLpro catalytic activity.***A*, chemical structure of famotidine. *B*, famotidine docked into SARS-CoV-2 PLpro catalytic site. *C*, famotidine docked into ISG15 binding site of SARS-CoV-2 PLpro. *D*, melting temperature of SARS-CoV-2 PLpro in the presence of famotidine or GRL-0617 in the T_M_ shift assay. *E*, *in vitro* PLpro inhibition assay. Initial velocity of AMC release from ubiquitin-AMC in different concentration of famotidine or GRL-0617 was measured and normalized against to control. *F*, effect of GRL-0617 and famotidine on SARS-CoV-2 PLpro activity to (*left*) ISG15-Cterm (ISG15c) or (*right*) ISG15 propargyl activity-based probes. Inhibitory effect of GRL-0617 and famotidine on ISG15 was tested with various concentration of GRL-0617 (0–100 μM) and famotidine (0–2 mM).

Several independent case studies on COVID-19 patients have suggested the use of this H2-receptor antagonist in the treatment of disease. Famotidine reduces the risk of intubation and death in hospitalized COVID-19 patients (1, 2). It has also been suggested to reduce respiratory symptoms in nonhospitalized patients (3). In combination with the H1-receptor antagonist cetirizine, it has been shown to reduce pulmonary distress in hospitalized COVID-19 patients (4). These studies indicated that famotidine has a beneficial role in managing COVID-19 disease symptoms, but the molecular basis of these observations remained elusive. The SARS-CoV-2 main protease (CoV-2 3CLpro) and Papain-like protease (SARS-CoV-2 PLpro) have been suggested to be the targets of famotidine based on several virtual screening studies (5, 6). However, *in vitro* studies did not show any inhibitory effect of famotidine on viral proteases (7).

The efficacy of famotidine in COVID-19 patients observed in several clinical studies makes it possible that famotidine may affect host pathways in response to viral infection. The histamine H2 receptor targeted by famotidine is not limited to the stomach, but is also found in the brain, the endocrine and exocrine glands, the pulmonary system, and the cardiovascular system. H2 receptors are also present on mast cells (MCs), which are deregulated in viral infections including those caused by coronaviruses (8, 9, 10). Studies show that famotidine (unlike cimetidine) reaches systemic concentrations that are sufficient to antagonize H2 receptors on other cell types such as those on MCs and neutrophils (9).

Deregulation of MCs contributes to a robust inflammatory response leading to a pulmonary cytokine storm that is seen in severe COVID-19 infections (11). Upon activation, MCs release proinflammatory cytokines and chemokines (IL-1, IL-6, IL-33, TNF, CCL2, MCP-1), histamine, prostaglandins, and leukotrienes. Increase in systemic histamine levels in combination with IL-1 causes inflammation-induced lung damage in SARS-CoV-2 infection (12, 13, 14). dsRNA virus activated MCs also trigger innate immune signaling through TLR3, which causes activation of IRF3 and synthesis of interferons (11).

TLR3-mediated signaling is an important antiviral signaling pathway that is activated in coronavirus infections (15). In humans, the TLR family comprises ten members (TLR1–TLR10), which are expressed in innate immune cells including macrophages, epithelial cells, and MCs. TLRs are pattern recognition receptors that recognize several pathogen-associated molecular patterns (PAMPs) present in bacteria, viruses, and other pathogens. TLRs on activation produce inflammatory cytokines, type I IFN, and other mediators. TLRs can be localized either on the cell surface (TLR-1, -2, -4, -5, -6, -10) or in endosomes (TLR-3, -7, -8, -9). SARS-CoV-2 enters the cell through the endosomal pathway and hence activates the endosomal TLRs. TLR activation *via* MyD88-dependent and TRIF-dependent pathways causes nuclear translocation of the transcription factors NF-κB, IRF-3, and IRF-7, with production of innate proinflammatory cytokines (IL-1, IL- 6, TNF-α) and type I IFN-α/β, which are essential for antiviral responses. TLR3-dependent signaling is an important innate immune response to coronaviral infections (16). Though TLR3 is beneficial in the initial viral clearance, hyperactivation may contribute to hyperinflammation and cause cytokine storms characteristic of severe cases of the disease (17).

In this study, we assess the effect of famotidine on viral proteases (SARS-CoV-2 3CLPro and PLpro) and host cells using computational docking, *in vitro* and cell biological assays and infection studies. Our studies show that on infection, famotidine does not affect the viral life cycle and replication, but affects host cells by histamine-induced signaling processes. In cells treated with poly(I:C), which mimics viral RNA, histamine increases TLR3 expression leading to an increase in the downstream IRF3 and NF-κB-dependent signaling. SARS-CoV-2-infected Caco2 cells pretreated with famotidine show reduced activation of IRF3/NF-κB and had lower mRNA levels of the interferon gene ISG15 and inflammatory mediator CCL2. The inhibitory effect of famotidine on TBK1/IRF3 signaling can be restored by pretreating cells with the TLR3 inhibitor CU CPT4a. These observations indicate a molecular basis of how on-target effects of famotidine may help in management of histamine-induced inflammation in severe COVID-19 patients.

## Results

### In *silico* docking studies show two potential binding sites in SARS-CoV-2 PLpro for famotidine

Since several studies suggested that SARS-CoV-2 PLpro may be a target of famotidine (5, 6), we checked for the possibility of famotidine binding to SARS-CoV-2 PLpro by docking famotidine to the SARS-CoV-2 PLpro protein structure (PDB ID: 7CJM). The results of docking showed two putative binding sites. At the catalytic site of SARS-CoV-2 PLpro, where naphthalene inhibitors such as GRL-0617 bind (Fig. 1*B*) (18, 19), the famotidine-binding mode is mainly electrostatic, dominated by three ionic interactions with R154, E165, and D162. This binding mode also shows three hydrogen bonds with the side chains of Y268, Q269, and T301, but lacks key interactions with P247 and P248 known from the GRL-0617-binding mode. The hydrogen bond between the sulfur of famotidine and the backbone of Q269 (S…NH) was far weaker than the CO…NH hydrogen bond in GRL-0617 binding. Interestingly, docking found another potential binding site near the S2 helix, which overlaps with the binding site for the N-lobes of ISG15 and K48-Ub2 (near F69 and T75) (Fig. 1*C*) (19, 20). Binding of famotidine in this site may thus interfere with the binding of ISG15. However, this binding mode is less promising because the only remarkable interactions were an ionic interaction with R65 and a suboptimal cation-π interaction with F69. The interactions with P59 and P75 were too weak to contribute significantly to binding affinity.

### Famotidine does not bind SARS-CoV-2 PLpro or affect its catalytic activity *in vitro*

To test whether famotidine binds to the SARS-CoV-2 PLpro *in vitro*, we employed a thermal shift assay. In this assay inhibitor binding is assessed by the shift in the melting temperature of the target protein, which correlates with the strength of interaction between the protein and the putative drug. We found that the previously confirmed SARS-CoV-2 PLpro inhibitor GRL-0617 increased the T_m_ of SARS-CoV-2 PLpro. By contrast, famotidine showed no effect on the denaturation curve of the protein (Fig. 1*D*). Similarly, famotidine did not affect the catalytic activity of either SARS-CoV-2 PLpro or SARS-CoV PLpro, measured by the velocity of AMC release from ubiquitin-AMC (ubiquitin–7-amido-4-methylcoumarin) in the presence of different concentrations of famotidine. On the other hand, GRL-0617 reduced the catalytic activity of both SARS-CoV-2

PLpro and SARS-CoV PLpro in the same assay (Fig. 1*E*). To examine whether famotidine can alter the binding of ISG15 to SARS-CoV-2 PLpro, we followed the modification of SARS-CoV-2 PLpro with ISG15-activity-based probes carrying a propargyl warhead. Whereas the level of modified SARS-CoV-2 PLpro decreased with GRL-0617, there were no such differences with famotidine (Fig. 1*F*). This result indicates that famotidine does not interact with PLpro or affect its activity *in vitro*.

### Lack of effect of famotidine on interferon signaling and NF-κB-dependent gene expression in SARS-CoV-2 PLpro expressing cells

To test whether famotidine can affect PLpro in transfected cells, we checked the interaction between ISG15 and the catalytically inactive SARS-CoV-2 PLpro in interferon-α-treated A549 cells. ISG15 was coimmunoprecipitated with the mutant SARS-CoV-2 PLpro in interferon-α-treated cells. GRL-0617 inhibited this interaction, but famotidine had no effect (Fig. S1*a*). Previous studies have shown an inhibitory effect of coronaviral PLproteases on the type I interferon response and on NF-κB signaling (19, 20). SARS-CoV-2 PLpro and SARS-CoV PLpro inhibit interferon signaling by inhibiting IRF3-dependent gene expression (19, 21, 22). Phosphorylation of TBK1 and IRF3 was inhibited in SARS-CoV-2 PLpro expressing A549 cells treated with interferon-α. GRL-0617 causes partial rescue of this inhibition, but famotidine has no effect (Fig. S1*b*). From existing literature and previous studies, it is known that SARS-CoV PLpro inhibits NF-κB signaling while SARS-CoV-2 PLpro exerts a stronger inhibitory effect on interferon signaling (18, 19). Famotidine treatment (4 h, 50 μM) had no significant effect on interferon-β and

NF-κB promoter activation in either SARS-CoV-2 PLpro or SARS-CoV PLpro transfected cells (Fig. S1, *c* and *d*). Also, famotidine did not affect the expression of cytokines, which are downstream of the type I interferon pathway (ISG15) or NF-κB-dependent inflammatory signaling (IL-6, IL-8) in SARS-CoV-2 PLpro expressing cells (Fig. S1*f*). Therefore, these experiments corroborate that famotidine does not show any effect on the interferon and NF-κB pathways in SARS-CoV-2 PLpro transfected cells.

### Famotidine has no effect on the main protease (3CLpro) or on viral replication

**Figure 2:**
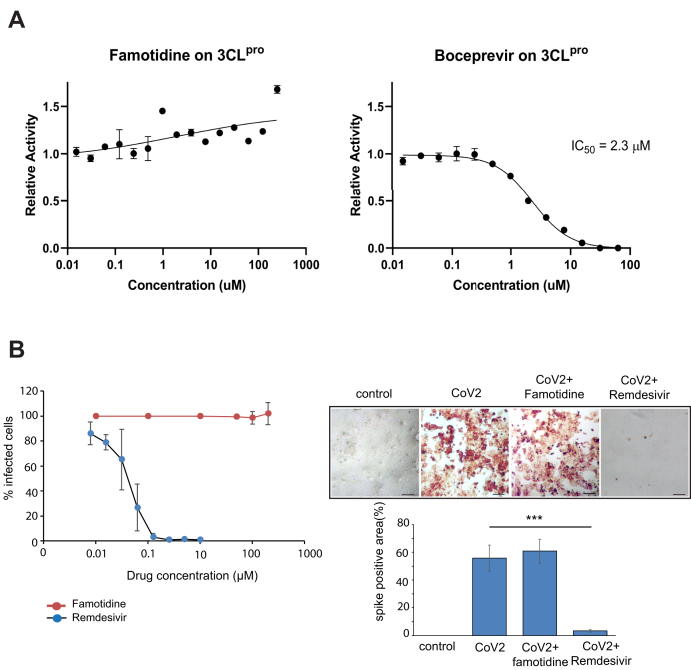
**Effect of famotidine on 3CLPro activity and viral replication.***A*, 3CLpro protein was preincubated with varying concentration of famotidine, followed by the addition of protein substrate as described in Experimental procedures section. Famotidine showed no inhibitory effect on CoV-2 3CLpro, when compared with positive control, Boceprevir, which inhibited the 3CLpro protease with an IC50 value of 2.3 μM. *B*, effect of famotidine and remdesivir on virus-infected cells after 24 h of infection, where remdesivir or famotidine was added at the time of infection. Spike positive area was measured 24 h after infection. Error bars indicate SD from three experiments. ∗∗∗*p* < 0.005. Scale bars: 100 μm.

Several reports suggest that the antiviral effect of famotidine on patients is due to inhibition of the coronavirus main protease (23). The main protease of SARS-CoV-2 is a cysteine protease that shares ~95% homology to the 3CLpro of SARS-CoV. It cleaves the viral polyproteins and is hence essential for viral replication. Boceprevir has been found to be one of the strongest inhibitors of SARS-CoV-2 3CLPro, inhibiting ~60% activity of the protease at a concentration of 20 μM in a fluorescence resonance energy transfer (FRET)-based protease activity assay (24). We observed a robust decrease in 3CL-pro activity upon boceprevir treatment with an IC_50_ of 2.3 ± 0.2 μM. On the other hand, famotidine did not inhibit the activity of CoV-2 3CLpro (Fig. 2*A*). We further checked viral replication in cells treated with famotidine or remdesivir. Remdesivir inhibits the RNA-dependent RNA polymerase of SARSCoV-2 (25, 26). There was no significant change in viral replication or any cytopathic effect (CPE) (measured by spike positive area) in famotidine-treated cells (Fig. 2*B*). Remdesivir caused a gradual dose-dependent inhibition of SARS-CoV-2-induced CPE with 1.25 μM remdesivir almost completely inhibiting CPE (Fig. 2*B*).

### Effect of famotidine on the proteome in SARS-CoV-2-infected cells

Since famotidine is an H2 receptor antagonist, we assumed that the beneficial effects of famotidine in patients may be due to its on-target effect on H2 receptors. To see a global effect of famotidine in SARS-CoV-2 infected cells, we analyzed the proteome of cells infected with SARSCoV-2 in the presence of histamine with or without famotidine treatment. Famotidine treatment led to a decrease in several proteins associated with the interferon pathway (IFIT1, IFIT2, ISG20, Herc5, Ube2L6), NF-κB pathway (NKAP, NFKBIB, RIPK1), and those associated to TLR signaling (TRAF6, MyD88) (Fig. S2, Table S1). Gene ontology and network analysis of the proteomic data using the Metascape software identified pathways associated with interferon response, cytokine production, viral infection and NF-KB signaling to be significantly altered by famotidine treatment (Fig. S3, *a* and *b*). Some proteins were also upregulated in famotidine-treated cells, though from the GO analysis these appear to be unrelated to viral infection (Fig. S3*c*). These results suggested that though famotidine does not affect viral replication, it may affect the antiviral response in infected cells and production of cytokines.

### Famotidine affects TLR3 expression in histamine-treated A549 cells

**Figure 3:**
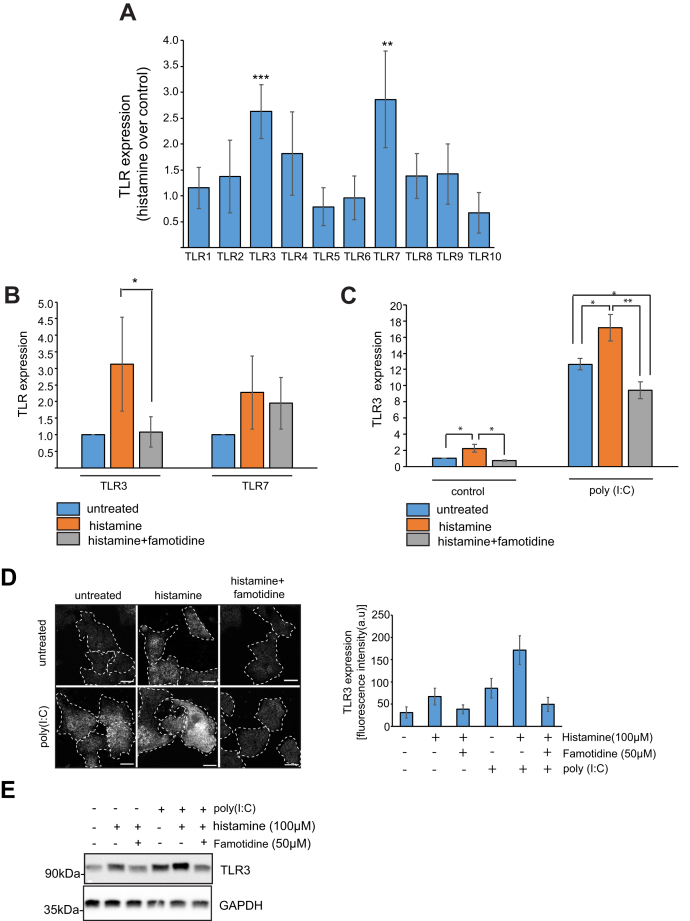
**Effect of histamine and famotidine on expression of TLRs**. *A*, A549 cells were treated with 100 μM histamine for 12 h followed by RT-PCR using TLR specific primers. Error bars indicate SD, ∗∗∗*p* < 0.005, ∗∗*p* < 0.01. *B*, mRNA expression of TLR3 and TLR7 from A549 cells treated with 100 μM histamine and 50 μM famotidine for 12 h. Error bars indicate SD, ∗*p* < 0.05. *C*, mRNA expression of TLR3 from A549 cells treated with 100 μM histamine and 50 μM famotidine for 12 h followed by treatment with poly(I:C) for 12 h. Error bars indicate SD, ∗∗*p* < 0.01, ∗*p* < 0.05. *D*, immunostaining of TLR3 in A549 cells treated with 100 μM histamine and 50 μM famotidine for 12 h followed by treatment with poly(I:C) for 12 h. Graph represents 30 cells taken from three independent experiments. Scale bars: 10 μm. *E*, immunoblotting of TLR3 in A549 cells treated with 100 μM histamine and 50 μM famotidine for 12 h followed by treatment with poly(I:C) for 12 h.

PCR amplification of histamine receptors from cDNA prepared from A549 cells showed that they expressed histamine H1 and H2 receptors and therefore may be used to monitor the on-target effect of histamine and famotidine on H2 receptors (Fig. S4*a*). Since toll-like receptors are an important arm of innate immune signaling, we decided to check the expression of TLRs in SARS-CoV-2-infected cells. Caco2 cells infected with SARS-CoV-2 for 24 h showed an upregulation of TLR3, TLR4, and TLR7 (Fig. S4*b*). Treatment of different cell types with histamine has been reported to upregulate expression of toll-like-receptor 3 (TLR3) (27, 28). In A549 cells, treatment with histamine for 12 h caused a modest upregulation of TLR3 and TLR7 mRNA levels (Fig. 3*A*). Pretreatment of these cells with famotidine reduced TLR3 mRNA levels, but did not affect TLR7 expression (Fig. 3*B*). To mimic the effect of viral RNA, cells were treated with poly(I:C) for 12 h and expression of TLRs was measured by RT-PCR. Treatment with poly(I:C) upregulated the expression of endosomal TLRs including TLR3, TLR7, TLR8, and TLR9 (Fig. S4*c*).

To mimic a scenario where the virus is present in high concentration of systemic histamine, we pretreated A549 cells with histamine and famotidine and then transfected them with poly(I:C). Pretreatment with histamine led to a significant increase in TLR3 levels, which was greater than that caused by poly(I:C) alone. This cumulative effect of histamine and poly(I:C) could be reversed when famotidine was present in the medium (Fig. 3*C*). Cell viability was not significantly affected by poly(I:C) treatment or by treatment with 100 μM histamine or 50 μM famotidine when measured with an MTT assay (Fig. S4*d*). The effect of histamine and poly(I:C) on TLR3 expression was also evident when endogenous TLR3 was immunostained with TLR3 antibody and in cell lysates immunoblotted for TLR3. Cells pretreated with histamine for 12 h prior to poly(I:C) treatment had the highest levels of TLR3, which was again rescued by famotidine treatment (Fig. 3, *D* and *E*). The effect of histamine on TLR3 expression is absent in TLR3 siRNA-treated cells (Fig. S4*e*). These results collectively show that poly(I:C) and histamine increase TLR3 levels, which can be reversed by famotidine.

### TLR3-dependent signaling in famotidine-treated cells

**Figure 4:**
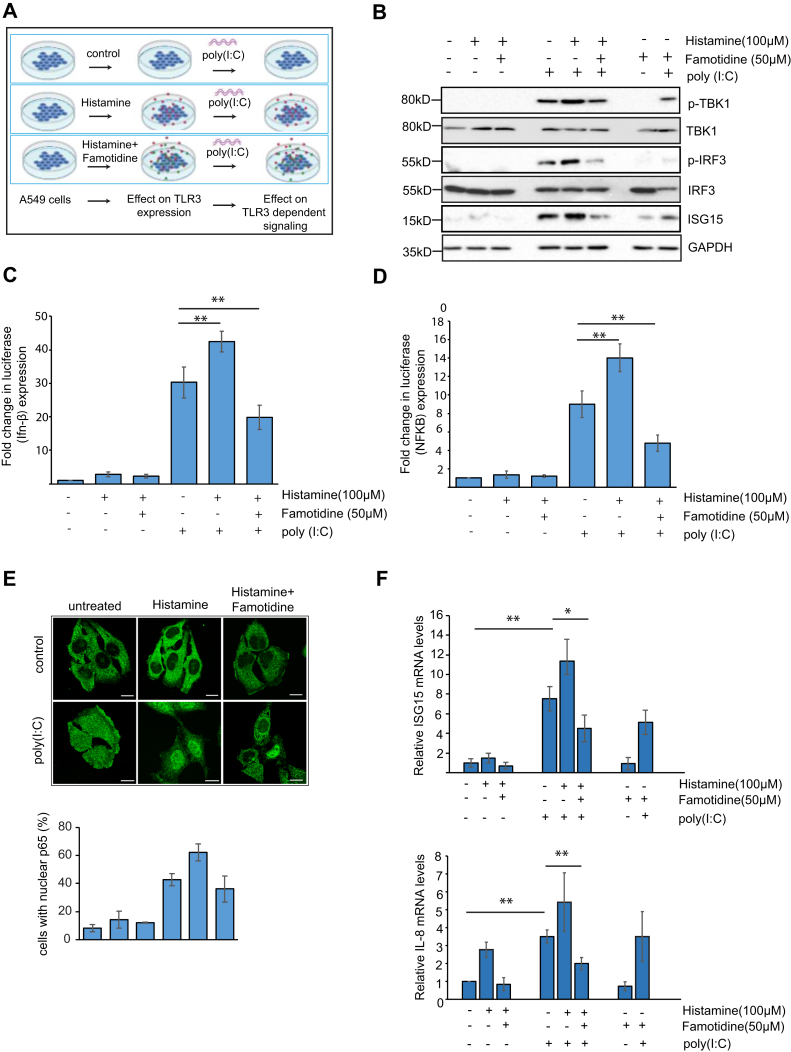
**Effect of histamine and famotidine on TLR3-dependent signaling**. *A*, schematic of experiment. Pretreatment of cells with 100 μM histamine and 50 μM Famotidine for 12 h followed by poly(I:C) treatment was used to assay TLR3-dependent signaling. *B*, phosphorylation of TBK1, IRF3, and ISG15 levels were measured in A549 cells after 12 h of poly(I:C) treatment. Cells were pretreated with 100 μM histamine and 50 μM famotidine for 12 h prior to poly(I:C) treatment. *C*, effect of histamine and famotidine pretreatment on luciferase (interferon-β) expression. A549 cells expressing indicated luciferase constructs were pretreated with histamine and famotidine for 12 h followed by 12 h of poly (I:C) treatment. Interferon-β expression was measured by using a luciferase reporter assay. Fold changes of luciferase expression are presented. Data are presented as mean ± SD. ∗∗*p* < 0.01. *D*, effect of histamine and famotidine pretreatment on luciferase (NF-κB) expression. A549 cells expressing indicated luciferase constructs were treated with poly (I:C) for 2 h to induce NFκB expression. Fold changes of luciferase expression are presented. Data are presented as mean ± SD. ∗∗*p* < 0.01. *E*, NF-κB (p65) was immunostained in cells treated with poly(I:C) for 1 h with or without histamine and famotidine pretreatment. Percentage of cells with nuclear p65 was plotted taking data from 30 cells from three independent experiments. Scale bars:10 μm. *F*, RT-PCR to detect ISG15and IL8 mRNA levels in poly(I:C) treated A549 cells, which were pretreated with histamine and famotidine. Error bars indicate SD, ∗∗*p* < 0.01.

Next we measured the effect of famotidine on TLR3 signaling. Cells were pretreated with histamine alone or in combination with famotidine and then treated with the TLR3 agonist poly(I:C) to activate TLR3-dependent signaling (Fig. 4*A*). Histamine alone was not enough to trigger TLR3 signaling though it can affect TLR3 expression. Poly(I:C) treatment activated TLR3 signaling causing phosphorylation of TANK binding kinase 1 (TBK1) and interferon regulatory factor 3 (IRF3) and a subsequent increase in interferon-stimulated gene 15 (ISG15). Pretreatment with histamine further increases the activation of the TBK1/IRF3 pathway. Famotidine counters this effect leading to a decrease in IRF3 activation and lower ISG15 levels (Fig. 4*B*). Histamine and poly(I:C) treatment for 12 h increased IFN-β promotor activation, which could be reversed by famotidine (Fig. 4*C*). A similar effect was also seen on NF-κB(p65) promoter activation after 2 h of poly(I:C) treatment (Fig. 4*D*). NF-κB activation was also measured in histamine and poly(I:C)-treated cells by measuring nuclear NF-κB(p65) levels by immunofluorescence. Histamine and poly(I:C) caused an increase in the fraction of nuclear p65, which is reduced on famotidine treatment. RT-PCR to detect gene expression of ISG15 and IL8, which are downstream of IRF3 and NF-κB, showed that famotidine could reduce levels of these markers in poly(I:C)-treated cells (Fig. 4*F*). Taken together, these results indicate that famotidine may affect interferon and inflammatory pathways, which are activated by viral dsRNA downstream of TLR3.

### Famotidine targets TLR3-dependent pathways in SARS-CoV-2 infection

**Figure 5:**
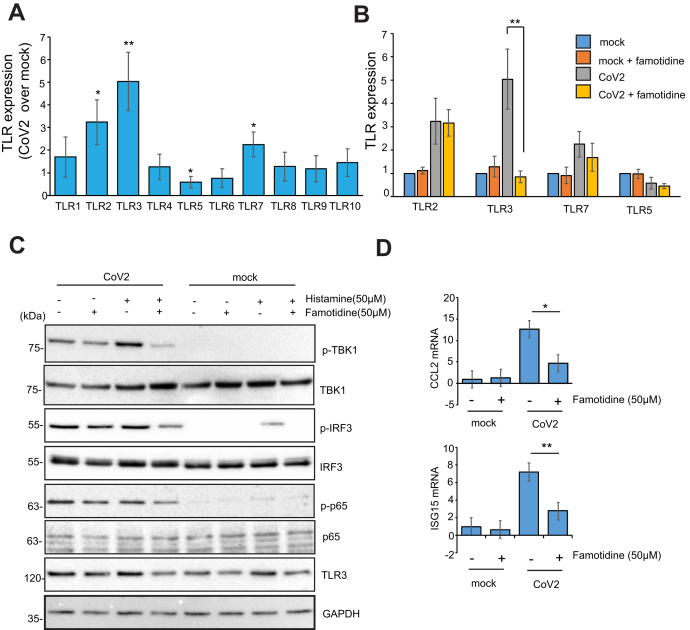
**Expression of TLRs and TLR3-dependent signaling in SARS-CoV-2infected cells**. *A*, Caco2 cells were infected for 24 h with SARS-CoV-2 after pretreatment with 100 μM histamine for 12 h. RNA was isolated with trizol, and RT-PCR was performed using TLR-specific primers. Error bars indicate SD, ∗∗*p* < 0.01, ∗*p* < 0.05. *B*, Caco2 cells infected with SARS-CoV-2 after pretreatment with histamine (12 h, 100 μM) and famotidine (50 μM, 12 h) were lysed in Trizol, followed by RNA isolation and RT-PCR using TLR specific primers. Error bars indicate SD, ∗∗*p* < 0.01. *C*, TLR3-dependent signaling was assayed after virus infection using indicated antibodies. *D*, CCL2 and ISG15 mRNA levels were measured by RT-PCR using RNA used in *panel B*.

Next we evaluated the effect of histamine on TLR3 expression in cells infected with SARS-CoV-2 infected Caco2 cells 24 h postinfection. Expression of TLR2, TLR3, and TLR7 was upregulated while TLR5 was downregulated in virus-infected cells compared with uninfected control (Fig. 5*A*). Famotidine treatment reduced TLR3 levels but did not affect the expression of the other TLRs (Fig. 5*B*). In our earlier study we had seen that viral infection causes phosphorylation of TBK1, IRF3, and NF-κB (p65). We also showed that GRL-0617 inhibits SARS-CoV-2 PLpro and releases the PLpro mediated inhibition on IRF3 and NF-KB signaling in SARS-CoV-2-infected cells (19). Here we showed that GRL-0617 treatment increased phosphorylation of TBK1 and IRF3 in CoV-2 infected cells, while famotidine caused a marginal decrease in phosphorylation of IRF3 and NF-κB (p65) (Fig. S5). Pretreatment of virus-infected cells with histamine increased the amount of phosphorylated TBK1 and IRF3. Famotidine counteracts this effect, reducing the phosphorylation of TBK1 and IRF3 significantly (Fig. 5*C*). Virus-infected cells also showed an increase in ISG15 and CCL2, which was reduced in cells pretreated with famotidine (Fig. 5*D*).

### CU-CPT 4a reverses the effect of famotidine on TBK1/IRF3 signaling in infected cells

**Figure 6:**
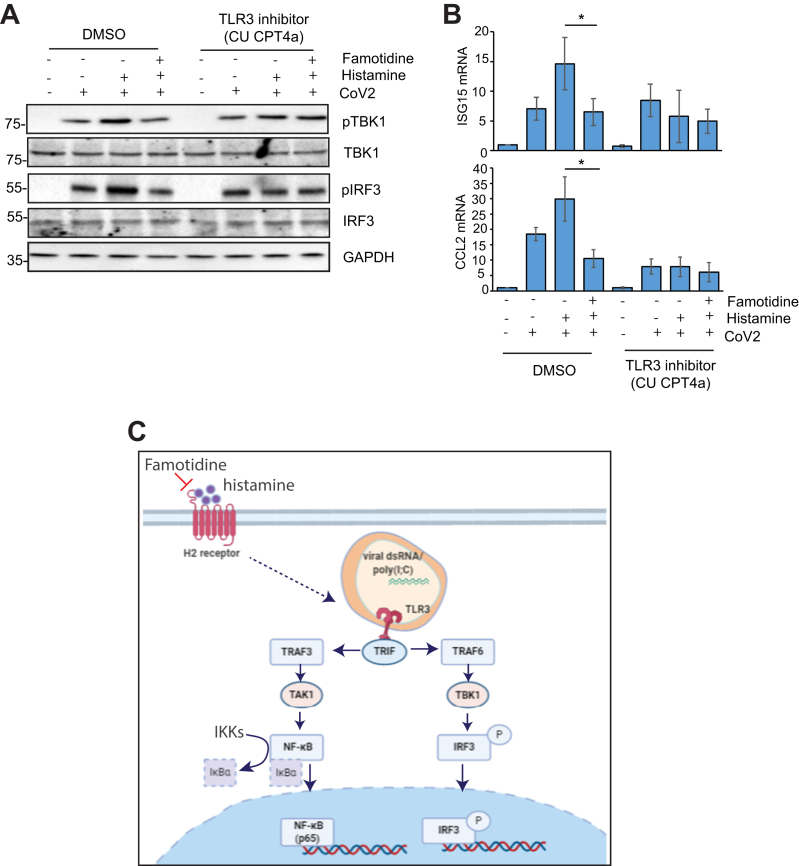
**Effect of TLR3 antagonist CU-CPT 4a on histamine-induced TLR3 signaling**. *A*, Caco2 cells were infected for 24 h with SARS-CoV-2 in presence of 10 μM CU-CPT 4a. Cells were pretreated with 100 μM histamine and 50 μM famotidine 12 h prior to infection. Lysates were immunoblotted using indicated antibodies. *B*, CCL2 and ISG15 mRNA levels were measured by RT-PCR using RNA isolated from infected cells treated with histamine, famotidine, and CU-CPT4a similar to panel *A*. Error bars indicate SD, ∗*p* < 0.05. *C*, summary figure showing the effect of famotidine on TLR3-dependent signaling.

To confirm that the effect of famotidine on the TBK1/IRF3 pathway is through TLR3-dependent signaling, we treated Caco2 cells with the TLR3 inhibitor CU-CPT 4a, which was added at the time of infection. TBK1 and IRF3 phosphorylation is unaffected by histamine and famotidine when TLR3 signaling is inhibited by CU-CPT 4a (Fig. 6*A*). Also, ISG15 and CCL2 levels are lower in the presence of the inhibitor and is unaffected by famotidine treatment (Fig. 6*B*). Therefore, at the molecular level, famotidine has cellular, not viral targets that influence, *e.g.*, the histamine-induced expression of TLR3 and, in turn, mediate innate immune signaling through the IRF3 and NF-κB pathway (Fig. 6*C*).

## Discussion

Many drugs are being repurposed to treat symptomatic COVID-19 patients and to decrease the chances of virus transmission. Several cohort studies of hospitalized COVID-19 patients showed a statistically significant reduced risk for death or intubation with famotidine-treated patients (1, 3, 4). Famotidine (Pepcid) scored well in computational studies where it was proposed to bind SARS-CoV-2 PLpro (5). However, *in vitro* assays showed that famotidine is not an antiviral drug. In cultured Vero E6 cells, famotidine did not inhibit viral replication (7). Our studies also confirmed that it does not affect SARS-CoV-2 proteases (3CL-Pro and PLpro) in *in vitro* as well as cell biological assays. It also had no effect on viral replication in cultured Caco2 cells. A recent report showed that a combination of famotidine with an H1 receptor antagonist cetirizine reduces pulmonary symptoms in COVID-19 patients (4). This suggested that the beneficial effects of famotidine may be due its effects on human cells and intracellular signaling controlling host immune responses.

Proteomic analysis of famotidine-treated cells infected with SARS-CoV-2 showed downregulation of proteins belonging to innate immune pathways such as interferon response, NF-κB, and cytokine production. Our study identified TLR signaling as a critical point of integration of famotidine treatment and innate immunity in SARS-CoV-2-infected cells. TLRs have been known to contribute to inflammatory lung damage in acute respiratory distress syndrome, which is often seen in severe COVID-19 patients. Both viral infection and histamine treatment upregulated TLR3 and TLR7 expression. The expression of TLR3 (but not TLR7) was sensitive to famotidine. An increase in TLR3 expression was accompanied with increased activation of TLR3-dependent pathways (IRF3 and NF-κB) in SARS-CoV-2-infected cells. This effect can be rescued by prior treatment of cells with famotidine. Notably, the effect of famotidine treatment was on TLR3 mRNA and protein expression is independent of viral infection and can be seen at a similar extent in uninfected cells. However, increased TLR3 protein level translates to altered activation of TBK1/IRF3 and synthesis of inflammatory cytokines and interferon only when the viral RNA (or poly (I:C)) is present to activate TLR3. Moreover, inhibiting TLR3 signaling with CU-CPT 4a in histamine-treated virus-infected cells eliminates the stimulatory effect of histamine on TBK1/IRF3 signaling and makes it insensitive to famotidine treatment. A slight decrease in IRF3 phosphorylation in infected cells treated with famotidine in the absence of histamine was observed (Fig. S5). Famotidine also alters cAMP levels and can increase signaling through the MAPK pathway by increasing phosphorylation of ERK 1/2 (29, 30). This may contribute to inhibition of TLR3 dependent pathways in famotidine-treated cells.

Our data and previous studies report a stimulatory effect of histamine treatment on TLR3 mRNA and protein expression, which can be reduced by famotidine treatment. A recent study on MCs treated with histamine suggests the Akt/MAPK pathway could be involved in this upregulation of TLR3 since the Akt inhibitor LY294002 could rescue the effects of histamine on IL13 and MCP1 production (28).

Famotidine has very high affinity for histamine H2 receptors (14 nM) and its pharmacokinetic parameters show that it can reach functionally relevant concentrations in blood. Therefore, it can affect MCs, neutrophils, and eosinophils in the blood, which also have H2 receptors. Coronavirus infection leads to mast cell activation through toll-like receptors (11). Activated MCs then secrete inflammatory cytokines such as IL-1, IL-6, and TNF-α as well as bronchoconstrictor mediators such as histamine, prostaglandin-D2, and leukotriene-C4. Taken together, our study shows the importance of histamine and famotidine in regulating TLR3 signaling in terms of gene expression and downstream pathways in cells infected by SARS-CoV-2. These findings are relevant in explaining how famotidine and other antihistamines may be effective in reducing mortality in severe COVID-19 patients.

## Experimental procedures

### Plasmids construction

SARS-CoV-2 PLpro Ubl domain (amino acids, 746–1060 of Nsp3 protein) from SARS-CoV-2 and SARS-CoV was cloned into pET28b for bacterial expression as described before (19). For mammalian expression, PLpros were cloned into pEGFP-C1 (clontech).

### Protein purification

SARS-CoV-2 PLPro and SARS-CoV PLpro were purified from *Escherichia coli* as described before (19). Briefly, BL21(DE3) *E. coli* competent cells (NEB) were transformed with plasmids and grown in LB medium to an OD600 of 0.6–0.8 at 37 °C. Protein production was induced by addition of 0.5 mM isopropyl-D-thiogalactopyranoside (IPTG) overnight at 18 °C. Lysates were incubated with TALON beads (Takara) pre-equilibrated with lysis buffer and nonspecific proteins were cleared with washing. Proteins were eluted with elution buffer (50 mM Tris-HCl, 150 mM NaCl, 250 mM imidazole, 2 mM DTT, pH 8.5). Eluted proteins were buffer exchanged to storage buffer (20 mM Tris-HCl, 100 mM NaCl, 2 mM DTT, pH 8.5) and used for *in vitro* experiments.

The expression and purification of 3CLpro protein and protein substrate protein were performed as described previously (29, 30). The sequence of SARS-CoV-2 3CLpro was obtained from GenBank (accession number: YP_009725301), codon-optimized, and ordered from GenScript. A C-terminal poly-histidine-maltose binding protein (His6-MBP) tag with two in-between Factor Xa digestion sites was inserted. The recombinant protein substrate His6 – CFP – TSAVLQ↓SGFRKM – YFP (where ↓ represents the cleavage site) was constructed, expressed, and purified as described (31, 32).

### Molecular docking

As docking target, we used the structural model of SARS-CoV-2 PLpro with bound GRL-067 from our previous simulation study (19, and PDB ID: 6W9C, rerefined by T. Croll). The model was energy minimized using GROMACS v2019.6 (33). The ligand, famotidine, was taken from ZINC database (34) and then minimized using the SwissDock server. The ligand was docked to the minimized PLpro using the SwissDock program with the full flexibility of the ligand (35). The whole surface of PLpro was screened during the docking run, resulting in 255 poses. These binding poses were clustered using the Jarvis−Patrick method with a cutoff of 3 Å as implemented in the g_cluster command of GROMACS v2019.6 (33).

### AMC probe-based kinetic assay

For determination of enzyme kinetics (kcat and Km), ubiquitin-AMC was used as substrate of PLpro and the release of AMC was measured by increase of fluorescence (excitation/emission, 360/487 nm) on a 384-well microplate reader (PHERAstar FSX, BMG Labtech). Five microliters of solution containing different concentration of K48-Ub2–AMC (76–0 μM) was aliquoted into 384-well plate, and reaction was initiated by addition of 5 μl of PLpro to the well. Initial velocities of AMC release were normalized to a standard curve, and the velocity *versus* substrate concentration plot was further analyzed by Michaelis–Menten enzymatic kinetics, using the kcat function with fixed value of total enzyme concentration as provided above. The experiment was repeated at least three times.

### ISG15 activity-based probe assay

PLpro proteins were diluted (2 μM final concentration) with activation buffer (25 mM Tris-HCl pH 7.5, 150 mM NaCl, 10 mM DTT) and incubated for 10 min at 25 °C, and the activity-based probes were diluted (0.2 mg ml^−1^ final concentration) in dilution buffer (50 mM Tris-HCl 7.5, 150 mM NaCl). The reaction mixture was prepared by mixing equal volume of activated PLpro proteins (2 μM) and ISG15-activity-based probe (0.2 mg ml^−1^). Samples were taken at different time points, and the reactions were quenched by the addition of SDS sample buffer. Samples were further analysed by SDS–PAGE and stained with a silver staining kit.

### Dynamic scanning fluorimetry (DSF) assay

A solution of 2 μM PLPro in assay buffer (50 mM Tris, 150 mM NaCl) was mixed 1:1000 with SYPRO Orange (Sigma). The compounds to be tested were added to a final concentration of 10 μM. In total, 20 μl of each sample was placed in a 96-well plate and gradually heated from 25 to 95 °C. Fluorescence was monitored using an Mx3005P real-time PCR instrument (Stratagene) with excitation and emission filters set to 465 and 590 nm, respectively. The experiment was performed in triplicate, and data analyzed with the MxPro software.

### In *vitro* 3CLpro inhibition assay

The inhibition assay was based on FRET using a fluorescent protein substrate previously developed for SARS-CoV 3CLpro 1,2 (27, 28). In total, 0.1 μM of purified SARS-CoV-2 3CLpro was preincubated with 2 μl of famotidine with varying concentration (0–250 μM in 2-fold dilution) in pH 6.5 buffer containing 20 mM HEPES pH 6.5, 120 mM NaCl, 0.4 mM EDTA, 4 mM DTT for 30 min at 30 °C. In total, 10 μM of the recombinant substrate was rapidly mixed to initiate the reaction, and the protease activity was measured by FRET with excitation and emission wavelengths of 430 nm and 530 nm, respectively, using a multiplate reader using CLARIOstar multiplate reader (BMG LABTECH) at 25 °C for 2.5 h. Boceprevir and 5% DMSO were served as positive control and negative control, respectively (22). The reduction of fluorescence at 530 nm was fitted to a single exponential decay to obtain the observed rate constant (kobs) using GraphPad Prism 7.0 (GraphPad Software, Inc). Relative activity of 3CLpro was defined as the ratio of kobs with inhibitors to that without. The relative inhibition concentration (IC50) value was determined by fitting the relative activity at different inhibitor concentration to a four-parameter logistics equation.

### Virus preparation

Virus was prepared as described before (19). SARS-CoV-2 strain FFM1 (accession no. MT358638) was isolated from travelers returning from Wuhan (China) to Frankfurt (Germany) using Caco-2 cells. Virus titers were determined as TCID50 per ml in confluent cells in 96-well microtiter plates.

### Sample preparation for mass spectrometry

Caco2 cells were pretreated with 100 μM histamine and 50 μM famotidine for 12 h followed by infection with SARS-CoV-2 for 24 h. Virus-infected cells were lysed in SDS-lysis buffer (50 mM Tris, 2% SDS, 10 mM TCEP, 40 mM chloroacetamide, protease inhibitor cocktail) heated to 95 °C for 10 min. Proteins were precipitated with acetone and resuspended in 8 M urea. Isolated proteins were digested with 1:50 w/w LysC (Wako Chemicals, cleaves at the carboxylic side of lysine residue) and 1:100 w/w trypsin (Promega, Sequencing-grade; cleaves at the carboxylic side of lysine and arginine residues) overnight at 37 °C after dilution to a final urea concentration of 1 M. Digests were then acidified (pH 2–3) using trifluoroacetic acid (TFA). Peptides were purified using C18 SepPak columns (Waters). Desalted peptides were dried and 25 μg of peptides was resuspended in TMT-labeling buffer (200 mM EPPS pH 8.2, 20% acetonitrile). Peptides were subjected to TMT labeling with 1:2 peptide TMT ratio (w/w) for1 h at room temperature. The labeling reaction was quenched by addition of hydroxylamine to a final concentration of 0.5% and incubation at room temperature for an additional 15 min.

### Mass spectrometric data acquisition

Samples were pooled in equimolar ratio and subjected to High pH Reversed-Phase Peptide Fractionation kit (Thermo Fisher Scientific) following the manufacturer's instructions. Eluted fractions were dried in SpeedVac and peptides were resuspended in 3% acetonitrile/0.1% TFA for liquid chromatography–mass spectrometry. All mass spectrometry data was acquired in centroid mode on an Orbitrap Fusion Lumos mass spectrometer hyphenated to an easy-nLC 1200 nano HPLC system with a nanoFlex ion source (Thermo Fisher Scientific). Resuspended peptides were separated on an Easy nLC 1200 (Thermo Fisher Scientific) and a 22-cm-long, 75-μm-innerdiameter fused-silica column, which had been packed in house with 1.9-μm C18 particles (ReproSil-Pur, Dr Maisch) and kept at 45 °C using an integrated column oven (Sonation). Peptides were eluted by a nonlinear gradient from 8 to 60% acetonitrile over 155 min followed by an increase to 95%B in 1 min, which was held for another 10 min. Full-scan MS spectra (350–1400 m/z) were acquired at a resolution of 120,000 at m/z 200, a maximum injection time of 100 ms, and an AGC target value of 4 × 10^5^. Up to 20 most intense precursors, with charge state in between 2 and 5, were isolated using a 0.7 Th window. MS/MS spectra were acquired with a maximum injection time of 50 ms, AGC target value of 1.5 × 10^4^, and fragmented using CID with a normalized collision energy of 35%. SPS-MS3 scans were done on the ten most intense MS2 fragment ions having an isolation window of 0.7 Th (MS) and 2 m/z (MS2). Ions were fragmented using NCE of 50% and analyzed in the orbitrap with the resolution of 50,000 at m/z 200, scan range 110–500 m/z, AGC target value 1.5 × 10^5^, and a maximum injection time of 120 ms.

### Mass spectrometric data analysis

Raw mass spectrometry data were analyzed with Proteome Discoverer (v.2.4, Thermo Fisher Scientific) using Sequest HT as a search engine and performing recalibration of precursor masses by the Spectrum RC-node. Number of missed cleavages permitted was 2. Mass tolerance of precursor ions is 7 ppm and mass tolerance of fragment ions is 0.5 Da. Fragment spectra were searched against the human reference proteome (“one sequence per gene,” 20,531 sequences, version March 2020) and protein sequences SARS-CoV-2 (14 sequences, version February 2020) downloaded from Uniprot in March 2020, as well as common contaminants as included in “contaminants.fasta” provided with the MaxQuant software (version 1.6.11). Static modifications were TMT at the peptide N-terminus and lysines as well as carbamidomethyl at cysteine residues, dynamic modifications were set as oxidation of methionine and acetylation at the protein N-terminus. Matched spectra were filtered with Percolator, applying a false discovery rate of 1% on peptide spectrum match and protein level. Reporter intensities were normalized to the total protein intensities in Proteome Discoverer, assuming equal sample loading and additionally by median normalization using the NormalyzerDE package. Statistically significant changes between samples were determined in Perseus (v.1.6.6.0). Data set was first filtered for contaminants and biological replicates were grouped as one. Proteins were further filtered for two valid values present in at least one group. Missing values were imputated from normal distribution of data using default settings. Significant candidates were chosen using two-sided *t* test with error-corrected *p*-values (0.01. FDR) and log2(fold change) value minimum of ±0.5. To avoid false-positive protein identification, ≥2 unique peptides identified within a single protein were used for further analysis. Network and gene ontology analysis was performed with statistically significant hits using the online Metascape software (36).

### Antiviral assay

Confluent layers of Caco-2 cells in 96-well plates were infected with SARS-CoV-2 FFM1 at a MOI of 0.01. Virus was added simultaneously with famotidine and incubated in MEM supplemented with 1% FBS with different drug dilutions. CPE was assessed visually 48 h after infection. Cells were fixed and stained with an antibody against the spike protein of SARS-CoV-2, followed by staining with a peroxidase-conjugated secondary antibody and the addition of substrate. Data for each condition were collected for at least three biological replicates.

### Cell lysis and western blotting

Cells were lysed using lysis buffer (50 mM Tris (pH 7.5), 150 mM NaCl, 1% Triton-X-100) for cellular assays, and virus-infected cells were lysed in hot SDS-lysis buffer (50 mM Tris, 2% SDS, 10 mM TCEP, 40 mM chloroacetamide, protease inhibitor cocktail). In total, 20 μg protein was loaded in 10% Tris-glycine gel, which was run at 150 V for 1.5 h, followed by transfer to PVDF membrane for 2 h, 300 mA, and immunoblotted with antibodies.

### Antibodies

We used the following antibodies and dilutions for this study: ISG15 (cat. no. HPA004627, Sigma Aldrich/Merck; 1:1000), GAPDH (cat. no. 2118, Cell Signaling Technology; 1:2000), GFP trap beads (cat no. gta-100, ChromoTek), GFP (cat. no. sc-9996, Santa Cruz Biotechnology; 1:2000), IRF3 (cat. no. 4302, Cell Signaling Technology; 1:2000), phospho-IRF3(Ser396) (cat. no. 4947, Cell Signaling Technology; 1:1000), TBK1 (cat. no. 3013, Cell Signaling Technology; 1:2000), pTBK1 (cat. no. 3300–1 Epitomics; 1:1000), NF-κB p65 (cat. no. 8008, Santa Cruz Biotechnology; 1:2000)

### Luciferase activity assay

To analyze the induction of IFN-β induced genes and NF-κB genes, a luciferase reporter assay was used in A549 cells. In brief, an expression construct containing the luciferase ORF and the IFN-β promoter (IFN-β-luciferase) or NF-κB promoter was transfected. In total, 100 ng plasmid was used per one well of a 12-well dish. All transfections were performed in triplicate and the average of three experiments is shown in figures. Sixteen hours after transfection, cells were treated with histamine and famotidine for 12 h. Following this 500 ng poly(I:C) was transfected using lipofectamine for 12 h (for assessing Ifn-β promoter induction) and for 1 h (for NF-κB induction) Luciferase expression was measured using the Luciferase Reporter Assay System (Promega). Fold change was calculated by taking untreated cells as 1.

### Immunofluorescence and confocal imaging

HeLa cells were treated with 100 μM histamine and 50 μM Famotidine for 12 h. This was followed by treatment with 500 ng poly(I:C) for 1 h. Cells were fixed with paraformaldehyde, blocked in 5% serum, and immunostained overnight at 4 °C with antibody against p65. Confocal imaging was performed using the Zeiss LSM780 microscope system. An Ar ion laser (for excitation of GFP at 488 nm), a He-Ne laser (for excitation of Alexa Fluor 546 nm) were used with a 63 × 1.4 NA oil immersion objective. Images were analyzed in FIJI to determine colocalization between DAPI and immunostained p65. Results are indicative of 50 cells taken from three independent experiments; error bars indicate standard deviation.

### Quantification of viral and cellular RNA

SARS-CoV-2 RNA from cell culture supernatant samples was isolated using Trizol according to the manufacturer's instructions. In total, 1 μg RNA was converted to cDNA using random hexamer primer using Thermo cDNA synthesis kit. RT-PCR was performed using EvaGreen (Biotium, Catalog no: 89138-982) and the Biorad CFX Connect Real-time PCR system.18S rRNA amplification was used for normalization. Forward (F) and reverse (R) primers used are as follows:

TLR1 (F: CAGTGTCTGGTACACGCATGGT, R: TTTCAAAAACCGTGTCTGTTAAGAGA), TLR2 (F: GGCCAGCAAATTACCTGTGTG, R: AGGCGGACATCCTGAACCT), TLR3 (F: CCTGGTTTGTTAATTGGATTAACGA, R: TGAGGTGGAGTGTTGCAAAGG), TLR4 (F: CAGAGTTTCCTGCAATGGATCA, R: GCTTATCTGAAGGTGTTGCACAT) TLR5 (F: TGCCTTGAAGCCTTCAGTTATG, R: CCAACCACCACCATGATGAG), TLR6 (F: GAAGAA GAACAACCCTTTAGGATAGC, R: AGGCAAACAAAATGGAAGCTT), TLR7 (F: CAACCA GACCTCTACATTCCATTTTGGAA, R:TCTTCAGTGTCCACATTGGAAAC), TLR8 (F: TTA TGTGTTCCAGGAACTCAGAGAA, R: TAATACCCAAGTTGATAGTCGATAAGTTTG) TLR9 (F: CCACCCTGGAAGAGCTAAACC, R:GCCGTCCATGAATAGGAAGC) TLR10 (F: GCCCAAGGATAGGCGTAAATG, R: ATAGCAGCTCGAAGGTTTGCC), ISG15 (F: GAGAGGCAGCGAACTCATCT, R: AGGGACACCTGGAATTCGTT) IL6 (F: GCAGAAAAAGGCAAAGAATC; R: CTACATTTGCCGAAGAGC),CCL2 (F: GTGCTGACCCCAATAAGGAA, R: TGAGGTGGTTGTGGAAAAGA). 18S rRNA (F: AGAAACGGCTACCACATCCA; R: CACCAGACTTGCCCTCCA)

### Statistical analysis

For Luciferase assays, five biological replicates were used per experiment. Each of these replicates were further divided into two technical replicates before performing the assay to improve signal/noise ratio. Fold change in luminescence was calculated based on the luminescence readout of control cells treated with TNF or IFN-α. Mean values of fold change were plotted in a bar graph in MS Excel where error bars indicate standard deviation. *p* values were calculated by two-tailed Student's *t* test in MS Excel. For real-time PCR experiments, five biological replicates were used per condition. ΔCq was calculated from the difference of Cq values between the gene of interest and that of 18SrRNA. ΔΔCq was calculated from the difference of ΔCq values between control and treated samples. Change in gene expression was calculated by calculating 2ˆ(-ΔΔCq). Mean values of five replicates were plotted in MS Excel. Error bars indicate standard deviation. *p* values were calculated by two-tailed Student's *t* test in MS Excel.

## Data availability

The mass spectrometry data used in our study has been submitted to the ProteomeXchange Consortium *via* the PRIDE partner repository with the data set identifier PXD025703. Any additional data supporting the analyses in the manuscript are available from the corresponding author upon reasonable request.

## Supporting information

This article contains supporting information.

## Conflict of interest

The authors declare that they have no conflicts of interest with the contents of this article.
