# Supplementary material for: Famotidine inhibits toll-like receptor 3-mediated inflammatory signaling in SARS-CoV-2 infection
Source: J Biol Chem. 2021 Jun 30;297(2):100925. doi: 10.1016/j.jbc.2021.100925 (PMC8241579; doi:10.1016/j.jbc.2021.100925)
Supplement: Figures S1–S5 [file mmc2.pdf]

Supporting Information

(Supplementary Figures and Legends)

Supplementary Figure Legends

Figure S1: Effect of Famotidine and GRL-0617 in PLpro transfected cells.

(a) Interaction between catalytically inactive PLpro and ISG15 in interferon-α treated A549 cells.

(b) Levels of indicated proteins in lysates from interferon-α treated A549 cells expressing GFP PLpro^{CoV2}. Cells were treated with Interferon-α for 36h. Famotidine or GRL-0617 was added in the medium 12h prior to cell lysis.

(c) Effect of PLpro on luciferase (interferon-β) expression. A549 cells expressing indicated luciferase constructs were transfected with indicated GFP-PLpro and treated with poly (I:C) to induce interferon-β expression. Fold changes of luciferase expression are presented. Data are presented as mean ± S.D. * $p < 0.05$.

(d) Effect of PLpro on luciferase (NF-κB) expression. A549 cells expressing indicated luciferase constructs were transfected with indicated GFP-PLpro and treated with TNF-α (20ng/ml,1h) to induce NF-κB expression. Fold changes of luciferase expression are presented. Data are presented as mean ± S.D. * $p < 0.05$, ** $p < 0.01$.

(e) GFP-PLpro^{CoV2} transfected cells were treated with TNF-α (10ng/ml, 2h) or treated with interferon-α (200 U/ml, 36 h) in presence or absence of Famotidine or GRL-0617 followed by RNA isolation, cDNA preparation and real time PCR using primers against IL6, IL8 or ISG15. Interferon-α treatment was used to activate ISG15 mRNA expression while TNF- -α treatment was used to test the expression of IL6 and IL8 genes. Data represents 6 biological replicates taken from 3 experiments, data are presented as mean ± S.D. * $p < 0.1$, ** $p < 0.01$.

Figure S2: Proteome of Famotidine treated cells upon SARS-CoV2 infection

Caco2 cells infected with SARS-CoV2 after pretreatment with histamine (12h,100μM) and Famotidine (50μM,12h) were lysed in SDS lysis buffer, followed by proteomic analysis of lysates from cells treated with and without of famotidine. Data was analyzed by Perseus. Volcano plot indicates proteins which are enriched and de-enriched in Famotidine treated cells. Number of biological replicates used in analysis=3.

Figure S3: Pathway analysis of proteome from Famotidine treated cells

(a) Proteins which were significantly reduced in the proteome of Famotidine treated cells ($p<0.01$) (from experiment in Fig S3) were subjected to network analysis using the online Metascape software.

(b) GO analysis of proteins which were significantly reduced in famotidine treated cells.

(c)GO analysis of proteins which are significantly upregulated in proteome of famotidine treated cells.

Figure S4: Expression of histamine receptors and TLRs

(a) cDNA from A549 cells was used as template to amplify histamine receptors (H1R, H2R, H3R, H4R) in a PCR for 30 cycles followed by running the reaction product in an 1% agarose gel.

(b) mRNA expression of TLRs were assayed by RT-PCR from cDNA prepared from A549 cells treated with 20µg/ml poly(I:C) for 12h. Error bars indicate s.d, p **<0.01, * p<0.05

(c) Expression of TLRs was assayed by RT-PCR using RNA isolated from CoV2 infected Caco2 cells 24h after infection. Error bars indicate s.d, p **<0.01, * p<0.05.

(d) Cell viability was assessed by MTT assay after treatment of A549 cells with increasing amounts of poly (I:C) in presence or absence of histamine/Famotidine pretreatment (left panel). Poly(I:C) treatment was given for 12h. For pretreatment histamine (100µM) and Famotidine(50µM) was used for 12h.

(e) TLR3 siRNA treated cells were treated with poly(I:C) for 12h with or without pretreatment with 100µM histamine and 50µM Famotidine.TLR3 levels were assayed by immunostaining with TLR3 antibody. Scalebars:10µm.

Figure S5: Effect of Famotidine and GRL-0617 on SARS-CoV2 infected cells

SARS-CoV2 infected Caco2 cells were treated with 50µM of Famotidine or GRL-0617 added at the time of infection. Lysates were immunoblotted with indicated antibodies.

Figure S1

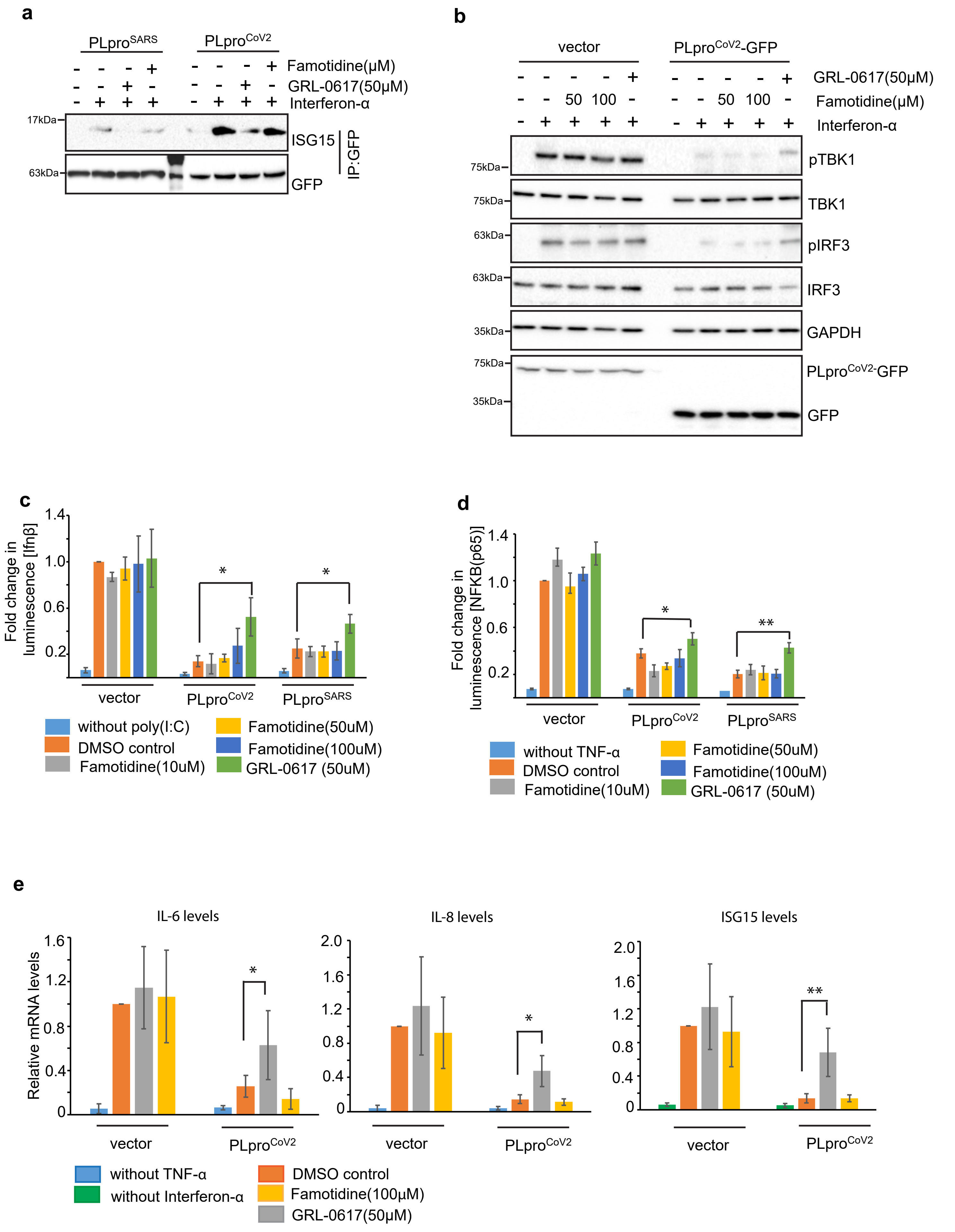

a
PLpro SARS
PLpro CoV2
Famotidine(μM)
GRL-0617(50μM)
Interferon-α
17kDa
63kDa
ISG15
GFP
IP:GFP
b
vector
PLpro CoV2-GFP
GRL-0617(50μM)
Famotidine(μM)
Interferon-α
75kDa
63kDa
35kDa
pTBK1
TBK1
pIRF3
IRF3
GAPDH
PLpro CoV2-GFP
GFP
c
Fold change in luminescence [Ifnβ]
without poly(I:C)
DMSO control
Famotidine(10uM)
Famotidine(50uM)
Famotidine(100uM)
GRL-0617 (50uM)
d
Fold change in luminescence [NFKB(p65)]
without TNF-α
DMSO control
Famotidine(10uM)
Famotidine(50uM)
Famotidine(100uM)
GRL-0617 (50uM)
e
IL-6 levels
IL-8 levels
ISG15 levels
Relative mRNA levels
vector
PLpro CoV2
without TNF-α
without Interferon-α
DMSO control
Famotidine(100μM)
GRL-0617(50μM)

Figure S2

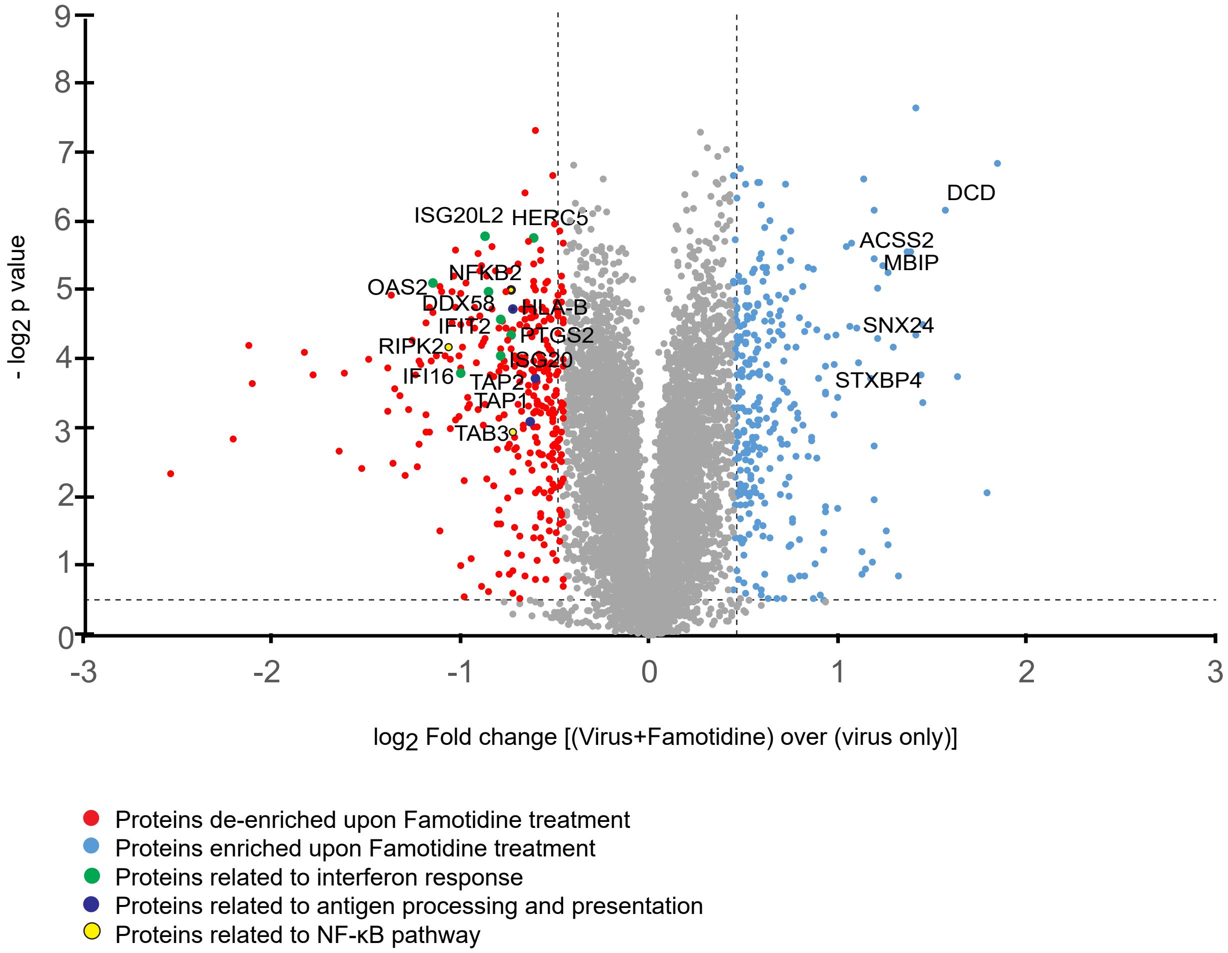

ISG20L2
HERC5
NFKB2
OAS2
DDX58
HLA-B
IFIT2
PTGS2
RIPK2
ISG20
IFI16
TAP2
TAP1
TAB3
DCD
ACSS2
MBIP
SNX24
STXBP4
- log2 p value
log2 Fold change [(Virus+Famotidine) over (virus only)]
Proteins de-enriched upon Famotidine treatment
Proteins enriched upon Famotidine treatment
Proteins related to interferon response
Proteins related to antigen processing and presentation
Proteins related to NF-κB pathway

Figure S3

(a)

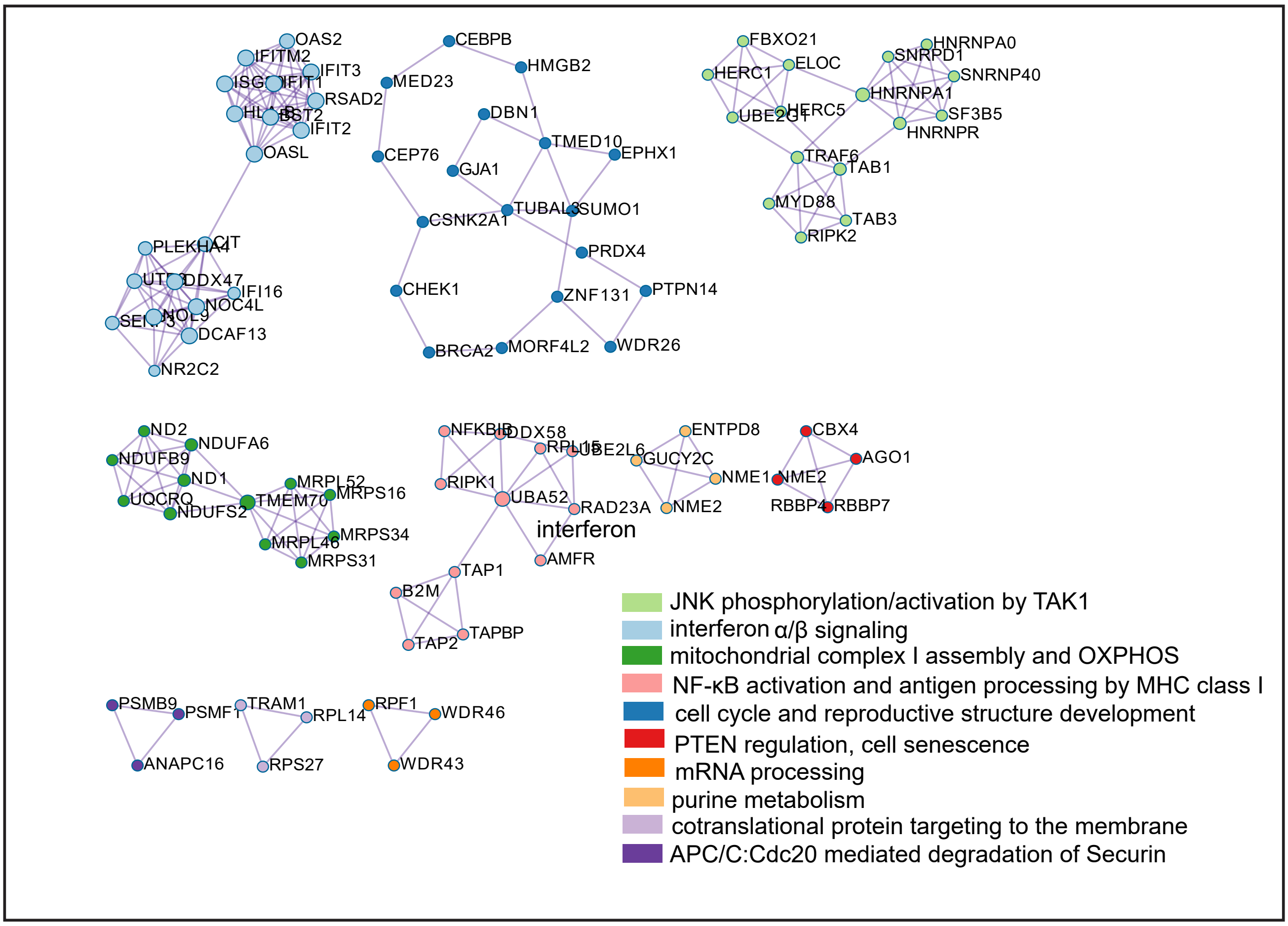

(b)

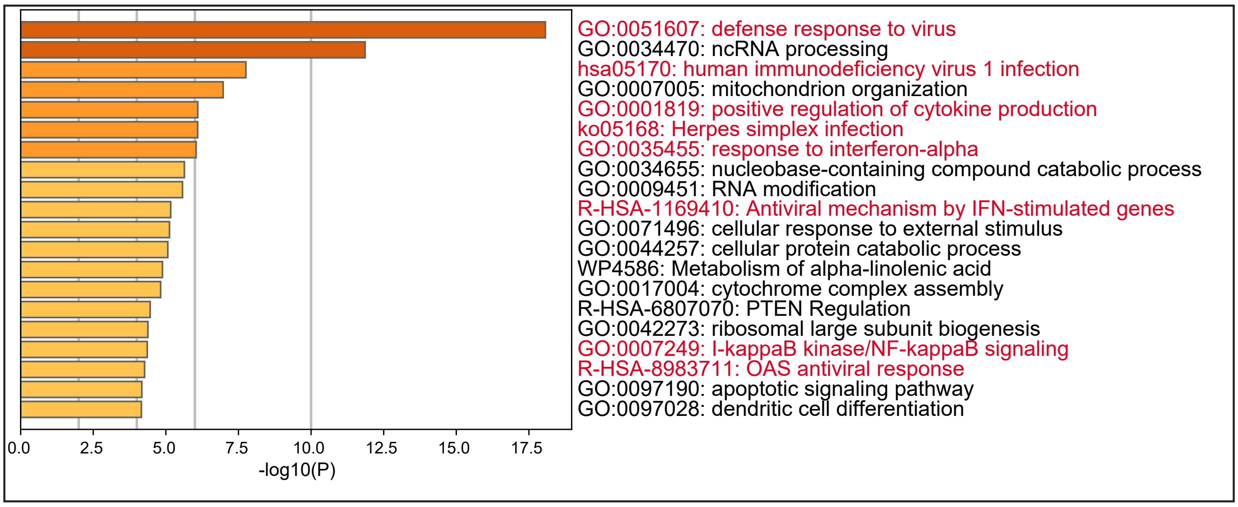

(c)

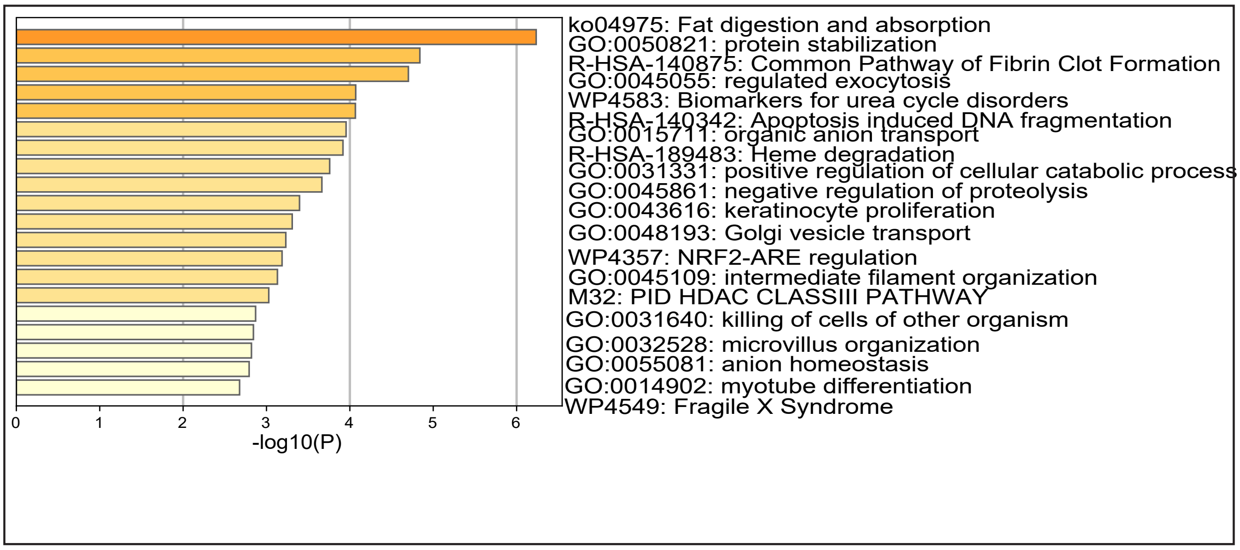

Figure S4

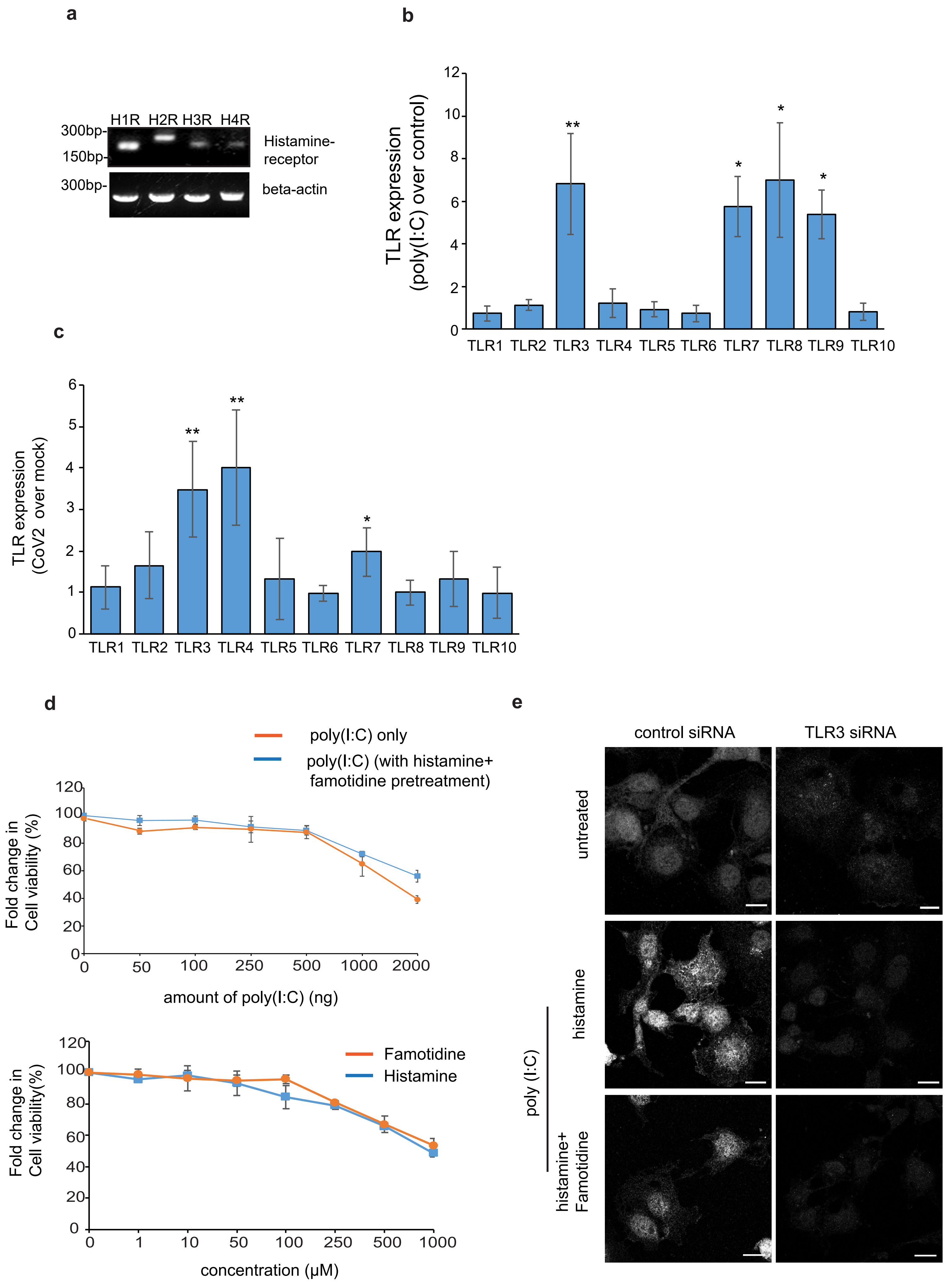
a
b
c
d
e
H1R H2R H3R H4R
300bp-
150bp-
300bp-
Histamine-receptor
beta-actin
TLR expression (poly(I:C) over control)
TLR expression (CoV2 over mock)
TLR1 TLR2 TLR3 TLR4 TLR5 TLR6 TLR7 TLR8 TLR9 TLR10
poly(I:C) only
poly(I:C) (with histamine+ famotidine pretreatment)
Fold change in Cell viability (%)
amount of poly(I:C) (ng)
Famotidine
Histamine
Fold change in Cell viability(%)
concentration (μM)
control siRNA
TLR3 siRNA
untreated
histamine
histamine+ Famotidine
poly (I:C)

Figure S5

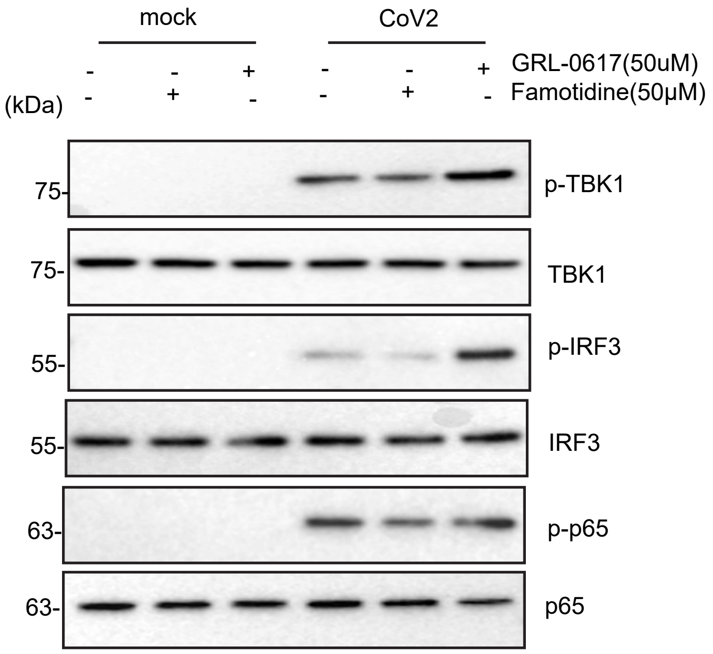
mock
CoV2
- - + - - + GRL-0617(50uM)
(kDa) - + - - + - Famotidine(50μM)
75-
p-TBK1
75-
TBK1
55-
p-IRF3
55-
IRF3
63-
p-p65
63-
p65
